# Early Surveillance and Public Health Emergency Responses Between Novel Coronavirus Disease 2019 and Avian Influenza in China: A Case-Comparison Study

**DOI:** 10.3389/fpubh.2021.629295

**Published:** 2021-08-10

**Authors:** Tiantian Zhang, Qian Wang, Ying Wang, Ge Bai, Ruiming Dai, Li Luo

**Affiliations:** ^1^School of Social Development and Public Policy, Fudan University, Shanghai, China; ^2^School of Public Health, Fudan University, Shanghai, China; ^3^Key Laboratory of Public Health Safety of the Ministry of Education and Key Laboratory of Health Technology Assessment of the Ministry of Health, Fudan University, Shanghai, China; ^4^Shanghai Institute of Infectious Disease and Biosecurity, School of Public Health, Fudan University, Shanghai, China

**Keywords:** COVID-19, emerging infectious diseases, H7N9, emergency management, China

## Abstract

**Background:** Since the novel coronavirus disease (COVID-19) has been a worldwide pandemic, the early surveillance and public health emergency disposal are considered crucial to curb this emerging infectious disease. However, studies of COVID-19 on this topic in China are relatively few.

**Methods:** A case-comparison study was conducted using a set of six key time nodes to form a reference framework for evaluating early surveillance and public health emergency disposal between H7N9 avian influenza (2013) in Shanghai and COVID-19 in Wuhan, China.

**Findings:** A report to the local Center for Disease Control and Prevention, China, for the first hospitalized patient was sent after 6 and 20 days for H7N9 avian influenza and COVID-19, respectively. In contrast, the pathogen was identified faster in the case of COVID-19 than in the case of H7N9 avian influenza (12 vs. 31 days). The government response to COVID-19 was 10 days later than that to avian influenza. The entire process of early surveillance and public health emergency disposal lasted 5 days longer in COVID-19 than in H7N9 avian influenza (46 vs. 41 days).

**Conclusions:** The identification of the unknown pathogen improved in China between the outbreaks of avian influenza and COVID-19. The longer emergency disposal period in the case of COVID-19 could be attributed to the government's slower response to the epidemic. Improving public health emergency management could lessen the adverse social effects of emerging infectious diseases and public health crisis in the future.

## Introduction

In the past 20 years, China has experienced several public health crises due to infectious disease outbreaks, such as severe acute respiratory syndrome in 2003, H1N1 swine influenza in 2009, and H7N9 avian influenza in 2013, seriously impacting health, economy, and global security (1–3). These outbreaks challenged the health emergency management in several countries, especially developing countries, including China (4, 5). In late December 2019, the novel coronavirus disease 2019 (COVID-19) emerged in Wuhan City, China, and rapidly spread worldwide (6). Prior to March 5, 2020, the Chinese government reported 80,409 confirmed cases and 3,012 fatalities due to COVID-19 (7).

COVID-19 and H7N9 avian influenza are two emerging infectious diseases that share similar characteristics (Table 1), such as probable development of severe respiratory diseases and susceptibility regardless of age. However, the socioeconomic losses were higher in COVID-19 outbreak than in H7N9 avian influenza. An effective public health emergency management reduces the adverse impact of emerging infectious diseases (8). This management relies on the early surveillance and timely information dissemination available in a given period (9). The following three key responses are often analyzed to evaluate the efficiency of public health emergency disposal: (1) time taken by the hospital to report an emerging infectious disease, (2) time taken to identify the pathogen, and (3) time taken by the government to respond (10–12). The World Health Organization declared a Public Health Emergency of International Concern on January 30, 2020 (13). Since then, China established and strengthened the national and local surveillance systems as well as emergency responses to prevent and control the spread of COVID-19 (14). Comparing the infectious disease surveillance and public health emergency disposal between different outbreaks in China could assist in improved public health strategies and decision-making by the government to prevent and control epidemics in the future, both in China and the world. To the best of our knowledge, few studies have been conducted to investigate the early disease surveillance and public health emergency disposal between other epidemics and COVID-19 in China.

**Table 1:** Characteristics of the H7N9 avian influenza and coronavirus disease 2019 in China.

**Characteristics**	**H7N9**	**COVID-19**
Country of origin	China	China
First case in China	February 2013 in Shanghai	December 2019 in Wuhan
Viral genome	Negative segmented RNA	Positive single-stranded RNA
Pathogen identification	CDC, China; March 29, 2013	CDC, China; January 7, 2020
Human-to-human transmission	Limited	High
Genesis/source	Domestic poultry	Unclear (so far)
Method of diagnosis in China	Real-time PCR	Real-time PCR
Vaccines in China	Not yet available	Not yet available

In this study, we aimed to conduct a retrospective study to compare the COVID-19 in Wuhan with the well-controlled H7N9 avian influenza (2013) in Shanghai, China, which should include the contents of the detection of the case, the initiation of emergency response, and etc. With the detailed comparison, the study would be able to summarize the lessons and propose measures to better improve the immediate responses to emergent public health events.

## Methods

### Data Collection

Data regarding the public health emergency disposal of the novel COVID-19 in Wuhan City, China, were obtained from published literature, secondary statistical data, WHO reports (3), official websites [e.g., National Health Commission of the People's Republic of China (http://en.nhc.gov.cn/), Chinese Center for Disease Control and Prevention (CDC) (http://www.chinacdc.cn/en/), Health Commission of Hubei Province, and Wuhan Municipal Health Commission], and credible media reports in China (CCTV, People's Daily, CBN, YiMagazine). Data regarding H7N9 avian influenza in Shanghai, China, were obtained from our published literature (15).

### Comparative Analysis

We compared the six key time nodes during the entire period from the detection of the first case to the launch of the health emergency response between COVID-19 in Wuhan City and H7N9 avian influenza in Shanghai. The key time nodes were as follows: hospitalization of the first case, hospital report to the local CDC, laboratory identification of the pathogen, technical recheck of the pathogen, confirmation, and notification of the pathogen, and launch of emergency disposal through the Chinese government.

We further evaluated three crucial periods during the public health emergency disposal of emerging infectious diseases: time taken by the hospital to report a case to the local CDC, time taken to identify the pathogen i.e., organization of the CDC laboratory to detect and recheck the pathogen, and time taken by the government to respond i.e., implementation of the emergency response once the pathogen is confirmed. Moreover, we calculated the number of days during each time node using the hospitalization time reference of the first case as the benchmark. The duration between detecting the first case and report the first death was also analyzed in the study.

The policy retrospective analysis approach was applied in this study, and no interviews, requiring recruitment and obtaining informed consent from humans were conducted. Information that can be disclosed to the public and/or is accessible in the public domain was sought in this study. Consequently, ethics approval was not required, and the study has no ethical implications associated with its design and conduct.

## Results

**Figure 1:**
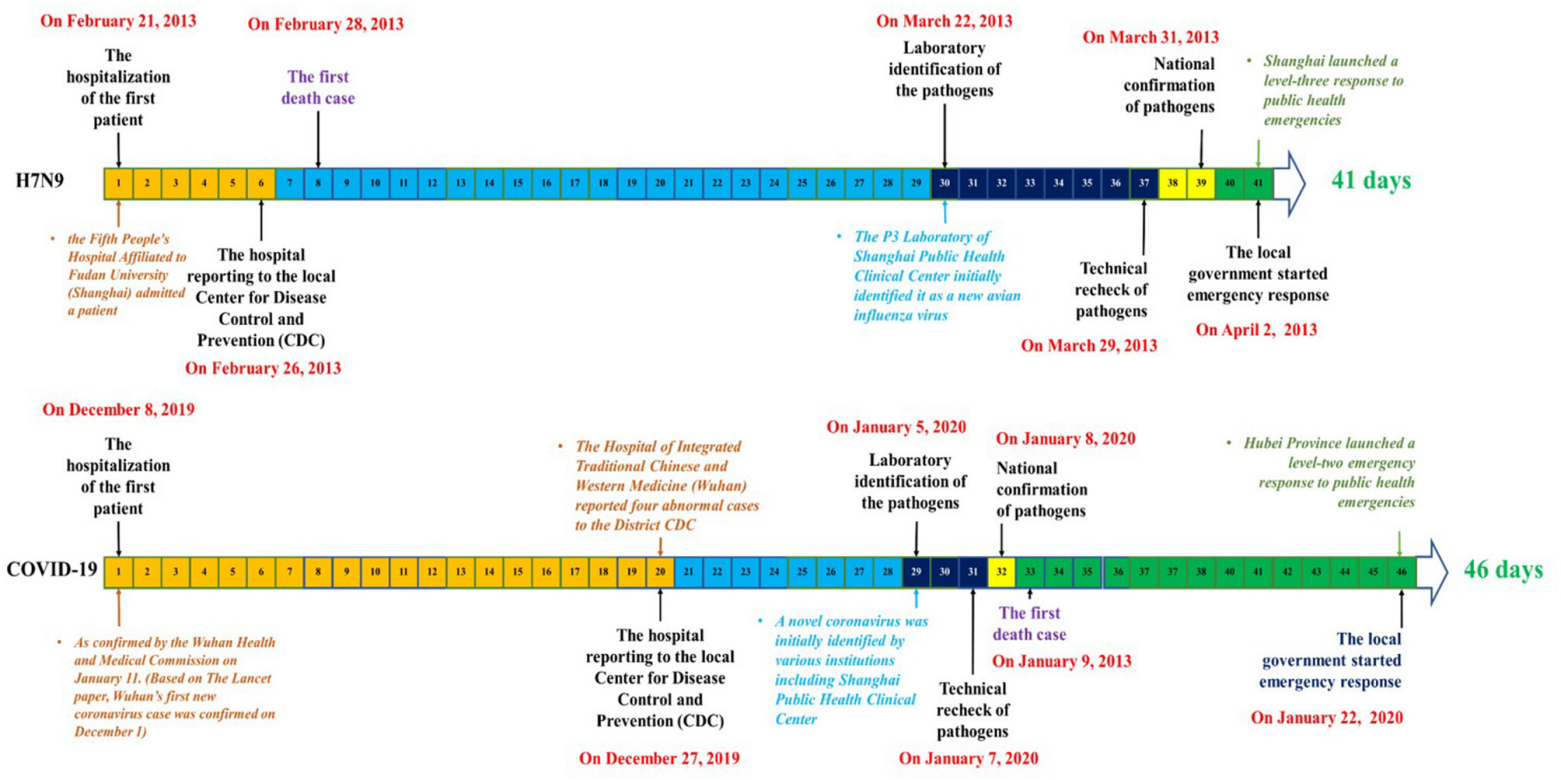
Comparison of the emergency disposal timeline between H7N9 avian influenza (2013) in Shanghai and coronavirus disease 2019 in Wuhan.

The comparison of three crucial periods between COVID-19 in Wuhan City and H7N9 avian influenza in Shanghai are shown in Table 2 and Figure 1. The entire process of early surveillance and public health emergency disposal was 5 days longer in the case of COVID-19 than in the case of H7N9 avian influenza (46 vs. 41 days). The details regarding the comparative analysis using the set of six key time nodes and three crucial time periods are as follows.

**Table 2:** Comparison of the key time nodes of emergency disposal between H7N9 avian influenza (2013) in Shanghai and coronavirus disease 2019 in Wuhan.

**Key time nodes**	**Three crucial periods**	**Shanghai H7N9 avian influenza (2013)**		**Wuhan novel coronavirus pneumonia (2019)**	
		**Dates and events**	**Cumulative time (day)**	**Dates and events**	**Cumulative time (day)**
1) Hospitalization of the first patient	Hospital to CDC reporting period	On February 21, the Fifth People's Hospital Affiliated to Fudan University (Shanghai) admitted a patient	1	On December 8, as confirmed by the Wuhan Health and Medical Commission on January 11 (based on *The Lancet* paper, Wuhan's first new coronavirus case was confirmed on December 1)	1
2) Hospital reporting to the local Center for Disease Control and Prevention (CDC)		On February 26, the Fifth People's Hospital Affiliated to Fudan University (Shanghai) submitted a report to the District CDC and requested for an epidemiological investigation	6	On December 27, the Hubei Hospital of Integrated Traditional Chinese and Western Medicine (Wuhan) reported four abnormal cases to the District CDC	20
3) Laboratory identification of the pathogen	Pathogen identification speed	On March 22, the P3 Laboratory of Shanghai Public Health Clinical Center initially identified it as a new avian influenza virus	30	On January 5, a novel coronavirus was initially identified by various institutions including Shanghai Public Health Clinical Center	29
4) Technical recheck of pathogen		On March 29, the National CDC isolated a new type of avian influenza virus from the patients' samples	37	On January 7, the National CDC isolated a novel coronavirus from the patients' samples	31
5) National confirmation of the pathogen	Government response period	On March 31, the National Health Administration confirmed that the pathogen was a new type of avian influenza virus	39	On January 8, the National Health Administration confirmed that the pathogen was a novel coronavirus	32
6) Local government launched emergency response		On April 2, Shanghai launched a level-three response to public health emergencies	41	On January 22, Hubei Province launched a level-two emergency response to public health emergencies	46

### Hospital to CDC Reporting Period

#### H7N9 Avian Influenza

The first patient was hospitalized at the Fifth People's Hospital of Shanghai affiliated to Fudan University on February 21, 2013. Subsequently, two patients were admitted (16, 17).

The doctor on duty in the emergency department observed that a paternal relationship existed between the follow-up case and the first case and believed that there was a possibility of transmission. Hence, in the early hours of February 26, 2013 at 1:10 a.m., he reported his findings to the doctor on duty who was also the chief of the infection department of the said hospital. He believed that the above situation was consistent with the possibility of clustered unexplained pneumonia cases and immediately called the attention of the administrators in charge of the hospital. Subsequently, the hospital gained expert consultation and undertook protective measures. At 2:30 a.m. of the same day, the hospital administrators contacted the chief administrative official of the local CDC by telephone and requested the start of epidemiological survey and sampling (18).

#### Coronavirus Disease 2019 (COVID-19)

The Wuhan Municipal Health Administration announced on January 11, 2020 that the first confirmed case of novel coronavirus pneumonia was detected on December 8, 2019 (18). A literature published in *The Lancet* reported that the first case was detected on December 1, 2019 (19). Based on the principle of caution, this article used December 8, 2019 as the onset time of the first case of the epidemic and considered that this patient was hospitalized in Wuhan Central Hospital at that time.

On the morning of December 26, 2019, Dr. Jixian Zhang, a doctor from Hubei Hospital of Integrated Traditional Chinese and Western Medicine in Wuhan City, observed an abnormality in a couple's lung computed tomography (CT) scan and an abnormality in their son's CT scan as well. The next day, the hospital reported four abnormal CT findings to the local CDC including another case (20).

Hence, the time taken by the hospital to report the first case of H7N9 (2013) in Shanghai and COVID-19 (2019) in Wuhan was 6 and 20 days, respectively.

### Pathogen Identification Period

#### H7N9 Avian Influenza

The local CDC conducted an epidemiological survey and sampling at 4:00 a.m. on February 26, 2013 and informed the hospital at 10:30 a.m. that adenovirus, syncytial virus, Legionella, H1N1, highly pathogenic avian influenza virus, Mycoplasma, and seasonal influenza virus tested negative. The hospital subsequently sent the samples to the P3 Laboratory of Shanghai Public Health Clinical Center. On March 22, the Shanghai Public Health Clinical Center preliminarily confirmed the pathogen as a new type of avian influenza virus. On March 29, 2013, the National CDC isolated a new type of avian influenza virus from samples collected from patients.

#### COVID-19

The local CDC was unable to identify the pathogen on December 26, 2019 and subsequently sent the samples to various testing institutions, including Shanghai Public Health Clinical Center and the Chinese Academy of Sciences (Wuhan Virus Institute). Various testing institutions had identified the novel coronavirus and the complete genome sequence between December 30, 2019 and January 5, 2020 (21). On January 7, 2020, the National CDC isolated a new type of coronavirus from the patients' samples (22).

Hence, the time taken to identify the pathogen in the cases of H7N9 (2013) in Shanghai and COVID-19 (2019) in Wuhan was 31 and 12 days, respectively.

### Government Response Period

#### H7N9 Avian Influenza

On March 31, 2013, the National Health Administration confirmed that the pathogen was a new type of avian influenza virus. On April 2, 2013, the government of Shanghai launched a level-three response (the emergency disposal work is leaded and directed by the Municipal government in its own administrative region) to public health emergencies.

#### COVID-19

On January 8, 2020, the National Health Administration confirmed that the pathogen was a novel coronavirus. On January 22, 2020, the government of Hubei Province launched a level-two response (the emergency disposal work is leaded and directed by the provincial Government within its administrative region) to public health emergencies (23).

Hence, the time taken by the government to respond in the cases of H7N9 (2013) in Shanghai and COVID-19 (2019) in Wuhan City was 4 and 14 days, respectively.

**Figure 2:**
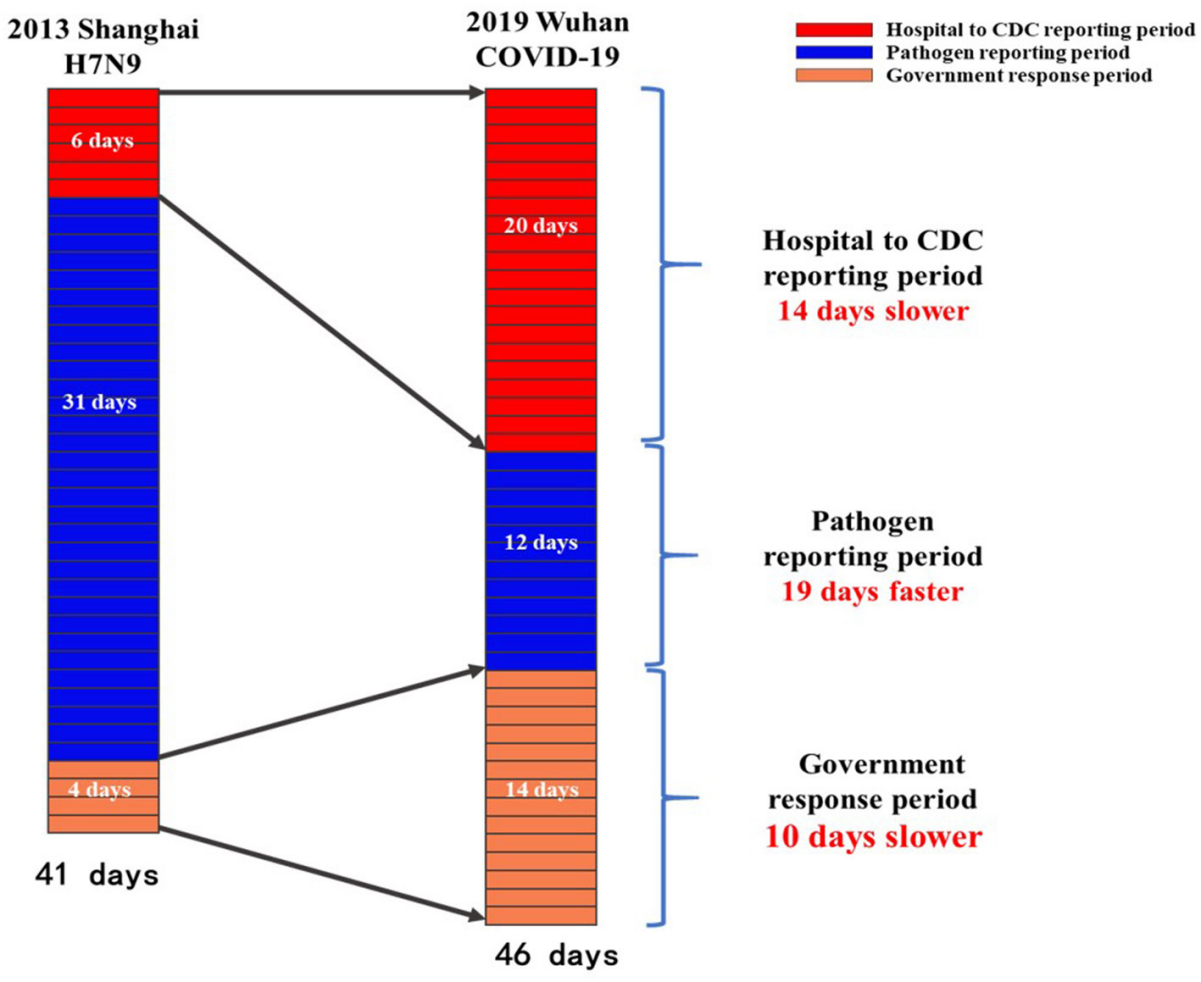
Comparison of three critical emergency disposal speed between H7N9 avian influenza (2013) in Shanghai vs. coronavirus disease 2019 in Wuhan.

We compared the government's emergency response process between outbreaks of Shanghai H7N9 avian influenza in 2013 and Wuhan COVID-19 in 2019. The time taken from the detection of the first case to the implementation of public health emergency response was 41 and 46 days for H7N9 avian influenza and COVID-19, respectively. The hospital to CDC reporting period was 14 days slower in the case of COVID-19 than in the case of H7N9 avian influenza. The time taken to identify the pathogen was 19 days faster in the case of COVID-19 than in the case of H7N9 avian influenza. Lastly, the time taken by the government to respond was 10 days slower in the case of COVID-19 than in the case of H7N9 avian influenza (Figure 2).

## Discussion

To the best of our knowledge, this was one of the few studies conducted in China to compare the strengths and weaknesses of public health emergency disposal between COVID-19 and H7N9 avian influenza. In this case-comparative study, the time taken to detect unknown pathogens had improved between the outbreaks of H7N9 avian influenza and COVID-19, whereas the time taken for hospitals to report a case to the local CDC and the government's emergency response was significantly increased.

In this study, we mainly investigated three crucial periods that influence the efficiency of emergency management of public health crises. During the emergency response process for H7N9 avian influenza (2013) in Shanghai, the maximum time was taken to technically identify and recheck the pathogen. The technical identification of pathogen took 24 days and the rechecking took 7 days, which accounted for 76% of the whole emergency process. In contrast, the time taken to technically identify and recheck the pathogen in the case of COVID-19 was reduced to just 12 days, accounting for 24% of the whole emergency process.

Laboratory identification was 19 days faster in the case of COVID-19 than in the case of H7N9 avian influenza, whereas the total disposal time was 5 days longer in the case of COVID-19 than in the case of H7N9 avian influenza. This could be attributed to the decrease in the reporting periods of certain hospitals and the increase in responding periods of the local governments. The time taken by the hospital to report a case to the local CDC was 14 days longer during COVID-19 than during H7N9 avian influenza (19 vs. 5 days, respectively). Furthermore, the response period of the local government launching emergency management was 14 days during COVID-19, which was 10 days longer than that during H7N9 avian influenza. Combining the hospital to CDC reporting period and government response period of H7N9 avian influenza with the pathogen identification period of COVID-19 would result in the entire epidemic control taking <22 days. Moreover, Hubei Province could thus launch an emergency response on December 30, suggesting that approximately 27 cases of COVID-19 would be detected in Hubei Province and the number of close contacts would be approximately 1350 by early March 2020. The Wuhan Municipal Infectious Diseases Hospital alone had 350 beds, which was sufficient to handle the full admission. Subsequently, the local CDC also had sufficient capabilities to screen and isolate most of the patients in close contacts with the infected patients.

The 5-day longer emergency period during COVID-19 could possibly be attributed to the hospital to CDC reporting period and government response period constrained by the following objective conditions: (1) At the beginning stage of the epidemic, H7N9 appeared a larger threat. The interval between the first identified case and the first reported death was only 7 days (on February 28, 2013, the first death case was observed). For COVID-19, this interval was 32 days instead. On January 9, local medical institutions and disease control departments were instructed to speed up and implement isolation and precautionary measures (20). (2) Because of underreporting of cases considering the challenges in data collection and shortage of testing kits and reagents in Hubei Province. Furthermore, the local medical supplies, beds, and facilities were insufficient, which were even exacerbated by the lockdown of the province, preventing the reach of supplies from several other hospitals.

Indeed, in addition to this research, we also carried out several other studies simultaneously, comparing the government emergency response time of COVID-19 with the SARS in 2003 and the pandemic influenza A (H1N1) in 2009 respectively. The study found that the emergency response time of the COVID-19 epidemic (46 days) was 18 days longer than that of H1N1 (28 days). The speed of hospital reporting, pathogen identification, and government decision-making of COVID-19 were all slower than those during HIN1 in particular. In the ongoing progress of the epidemic, the peak onset of H1N1 was about 4 weeks later than COVID-19, and the epidemic curve of H1N1 was flatter, which might be related to the timely emergency response of the HIN1 epidemic. The other study which compared the emergency response time between the SARS epidemic (127 days) and COVID-19, found that the latter was 81 days shorter. The hospital report time of these two epidemics was similar, but the pathogen detection time of SARS was more than 3 months longer than that of COVID-19, which reflected the level of pathogen detection in China has been greatly improved these years. After then, in following research, we will summarize the correlation between disposal time in different epidemics and their spread speed, attempt to explore the standard of emergency response procedures and their time constraints, so as to provide a reference for public health emergency response in the future.

This study has several potential limitations. First, the assessment coverage was at the city level; thus, comparison between the national level and the grassroots level was not assessed in this study. The grassroots level is the first gateway of public health emergency, and the effective measures and emergency responses taken by the grassroot level are considered important. Second, we used six-time nodes to evaluate the process of the government's emergency response, which is relatively limited when evaluating the possibility of an epidemic of major infectious diseases. Third, the data were based on China's official and authoritative reports, coupled with retrospective studies, which inevitably had information bias. Considering all these limitations, the findings should be interpreted with caution before additional studies are conducted.

## Conclusions

The identification of the unknown pathogen has significantly improved in China between the outbreaks of H7N9 avian influenza and COVID-19. However, the speed of the hospital reporting an emerging infectious disease and the speed of the government decision-making were slow in COVID-19 epidemic, which might be one of the vital factors for widespread COVID-19 cases. These issues need to be addressed urgently to prepare for public emergencies to prevent and control future epidemics of emerging infectious diseases in China and the world.

## Data Availability Statement

The original contributions presented in the study are included in the article/supplementary material, further inquiries can be directed to the corresponding author/s.

## Author Contributions

TZ, QW, and LL designed the project, processed and analyzed the data, and wrote the manuscript. YW, GB, and RD edited the manuscript. All authors revised the draft.

## Conflict of Interest

The authors declare that the research was conducted in the absence of any commercial or financial relationships that could be construed as a potential conflict of interest.

## Publisher's Note

All claims expressed in this article are solely those of the authors and do not necessarily represent those of their affiliated organizations, or those of the publisher, the editors and the reviewers. Any product that may be evaluated in this article, or claim that may be made by its manufacturer, is not guaranteed or endorsed by the publisher.

## References

[1] Li Z Gao GF. Infectious disease trends in China since the SARS outbreak. Lancet Infect Dis. (2017) 17:1113–5. 10.1016/S1473-3099(17)30579-0 29115254 PMC7128956

[2] Al-Tawfiq JA Zumla A Gautret P Gray GC Hui DS Al-Rabeeah AA. Surveillance for emerging respiratory viruses. Lancet Infect Dis. (2014) 14:992–1000. 10.1016/S1473-3099(14)70840-0 25189347 PMC7106459

[3] Rudge JW Coker R. Human to human transmission of H7N9. BMJ. (2013) 347:f4730. 10.1136/bmj.f4730 23920349

[4] Yu H Cowling BJ Feng L Lau EH Liao Q Tsang TK. Human infection with avian influenza A H7N9 virus: an assessment of clinical severity. Lancet. (2013) 382:138–45. 10.1016/S0140-6736(13)61207-6 23803487 PMC3801178

[5] Zhong NS Zheng BJ Li YM Poon Xie ZH Chan KH. Epidemiology and cause of severe acute respiratory syndrome (SARS) in Guangdong, People's Republic of China, in February 2003. Lancet. (2003) 362:1353–8. 10.1016/S0140-6736(03)14630-2 14585636 PMC7112415

[6] Hui DS Azhar EI Madani TA Ntoumi F Kock R Dar O. The continuing 2019-nCoV epidemic threat of novel coronaviruses to global health - The latest 2019 novel coronavirus outbreak in Wuhan, China. Int J Infect Dis. (2020) 91:264–6. 10.1016/j.ijid.2020.01.009 31953166 PMC7128332

[7] National Health Commission of the People's Republic of China. Guideline for National Surveillance, Excluding and Management on Unknown Etiology Pneumonia [EQ/OL]. (2004). Available online at: http://www.nhc.gov.cn/wjw/zcjd/201304/ad9b232676bb4671a20b6fbdd26c1376.shtml (accessed March 5, 2020)

[8] Verikios G Sullivan M Stojanovski P Giesecke J Woo G. Assessing regional risks from pandemic influenza: a scenario analysis. World Econ. (2016) 39:1225–55. 10.1111/twec.12296

[9] Qiu W Chu C Hou X Rutherford S Zhu B Tong Z. A comparison of China's risk communication in response to SARS and H7N9 using principles drawn from international practice. Disaster Med Public Health Prep. (2017) 12:587–98. 10.1017/dmp.2017.114 28974284

[10] Health MO. Guideline for National Surveillance, Excluding and Management on Unknown Etiology Pneumonia. Beijing (2007).

[11] Xiang N Havers F Chen T Song Y Tu W Li L. Use of national pneumonia surveillance to describe influenza A(H7N9) virus epidemiology, China, 2004–2013. Emerg Infect Dis. (2013) 19:1784–90. 10.3201/eid1911.130865 24206646 PMC3837642

[12] Xiang N Song Y Wang Y Wu J Millman AJ Greene CM. Lessons from an active surveillance pilot to assess the pneumonia of unknown etiology surveillance system in China, 2016: the need to increase clinician participation in the detection and reporting of emerging respiratory infectious diseases. BMC Infect Dis. (2019) 1:770. 10.1186/s12879-019-4345-0 31481020 PMC6724368

[13] WHO. Statement on the second meeting of the International Health Regulations (2005). Emergency Committee regarding the outbreak of novel coronavirus (2019-nCoV). (2020). Available online at: https://www.who.int/news-room/detail/30-01-2020-statement-on-the-second-meeting-of-the-international-health-regulations-(2005)-emergency-committee-regarding-the-outbreak-of-novel-coronavirus-(2019-ncov) (accessed March 5, 2020).

[14] Lu X Xue L. Managing the unexpected: sense-making in the Chinese emergency management system. Public Admin. (2016) 94:414–29. 10.1111/padm.12261

[15] Luo L Tian J Li F Zhou Y Jin C Wen W. Suggestions of improving epidemic pathogen detection based on the experience of H7N9 in Shanghai. China Health Rescour. (2014) 17:337–9. 10.3969/j.issn.1007-953X.2014.05.007

[16] Gao R Cao B Hu Y Feng Z Wang D Hu W. Human infection with a novel avian-origin influenza A (H7N9) virus. N Engl J Med. (2013) 368:1888–97. 10.1056/NEJMoa1304459 23577628

[17] Chen Y Liang W Yang S Wu N Gao H Sheng J. Human infections with the emerging avian influenza A H7N9 virus from wet market poultry: clinical analysis and characterisation of viral genome. Lancet. (2013) 381:1916–25. 10.1016/S0140-6736(13)60903-4 23623390 PMC7134567

[18] Wuhan Municipal Health Commission. Experts Interpret the Latest Notice of Viral Pneumonia of Unknown Causes. (2020). Available online at: http://wjw.wuhan.gov.cn/gsgg/202004/t20200430_1199592.shtml (accessed March 5, 2020).

[19] Huang C Wang Y Li X Ren L Zhao J Hu Y. Clinical features of patients infected with 2019 novel coronavirus in Wuhan, China. Lancet. (2020) 395:497–506. 10.1016/S0140-6736(20)30183-5 31986264 PMC7159299

[20] Chen R Xu BJ. If the Alarm Bell in Wuhan Has a Chance to be Sounded, What Day Can It be. YiMagazine (2020). Available online at: https://www.yicai.com/news/100495596.html (accessed March 5, 2020)

[21] Health Commission of Hebei Province. China's response to COVID-19 Gains International Recognition. [EB/OL] (2020).

[22] CCTV. Novel Coronavirus! Preliminary Determination of the “Culpri” of Unexplained Pneumonia in Wuhan [EB/OL]. (2020). Available online at: http://m.news.cctv.com/2020/01/09/ARTI9Vp9Lra4Tvltz3r7es96200109.shtml (accessed March 5, 2020).

[23] Hubei Provincial Government. Circular of Hubei Provincial People's Government on Strengthening Pneumonia Prevention and Control of New Coronavirus Infection [EB/OL]. (2020). Available online at: http://www.gov.cn/xinwen/2020-01/22/content_5471772.htm (accessed March 5, 2021).

